# Quality of Life in Hemodialysis Versus Peritoneal Dialysis Patients in Bahrain

**DOI:** 10.7759/cureus.49408

**Published:** 2023-11-25

**Authors:** Hend Aljenaidi, Lamees Alayoobi, Wejdan Alqassab, Ali Alfehaid, Madhawi Albuainain, Rahaf AlMuhanadi, Saad Alotaibi, Manar Almutiri, Ahmed Jaradat, Amgad E El-Agroudy

**Affiliations:** 1 Nephrology, Salmaniya Medical Complex, Manama, BHR; 2 Internal Medicine, Arabian Gulf University, Manama, BHR; 3 Family and Community Medicine, Arabian Gulf University, Manama, BHR

**Keywords:** chronic kidney disease, questionnaire, peritoneal dialysis, hemodialysis, quality of life

## Abstract

Introduction: Chronic diseases, including chronic kidney disease, affect patients' quality of life (QOL). Hemodialysis (HD) and peritoneal dialysis (PD) are renal replacement methods in these patients. This work aimed to study the relationship between QOL scores in patients with end-stage renal disease (ESRD) on HD and PD.

Methods: This study was done at Salmaniya Medical Complex (SMC), Bahrain, from May to July 2023. A standard QOL index score instrument in Arabic form was used on 76 HD and 38 PD patients. The inclusion criteria included dialysis for at least three months and an age of more than 18 years with no severe morbidities or psychological diseases.

Results: The mean age of HD and PD patients was 58.7 ± 11.2 and 55.9 ± 12.1 years, respectively. Thirty-five (46.1%) of the HD patients and 17 (44.7%) of the PD patients were females. In most dimensions, the QOL score of the patients treated with PD was better than that of the HD group. The number of hospital admissions was statistically significantly higher in the HD group (p = 0.007); however, there was no significant difference in the causes of admissions (p = 0.131). In this study, we observed the highest QOL score in the family subscale (93.2 ± 9.2 and 98.6 ± 4.7), followed by the psychological/spiritual subscale (81.1 ± 16.7 and 97.6 ± 3.9) in the HD and PD groups, respectively), but it was statistically significantly higher in the PD group (p < 0.001).

Conclusion: Our findings show that patients starting PD had better QOL scores in all domains than patients starting HD. Moreover, patients on PD maintained more active social support and ultimately felt better emotional well-being and physical health than those undergoing HD.

## Introduction

Chronic kidney disease and, hence, end-stage renal disease (ESRD) are growing worldwide, affecting >10% of the population worldwide, amounting to >800 million individuals, and have emerged as among the leading causes of mortality worldwide. Published statistics from the United States National Centre for Kidney Disease estimate that approximately 15% of the population suffer from CKD. These data show that 65% of the 600,000 suffering from this disease are on dialysis. This number is increasing at an alarming percent of approximately 8-10% annually [1]. Patients on dialysis are in a situation of abject dependence on a machine or a procedure, which is stressful for them for the rest of their lives. Most dialysis patients who are employed may seldom return to full-time work activity; have some financial worries, flexibility with time, a sense of security, and the ability to travel and sleep; and may suffer from anxiety and/or depression. These patients may have uncomfortable physical side effects that often come with the dialysis treatment, such as fatigue, nausea and vomiting, dizziness, difficulty sleeping, and loss of sex drive, and they can definitely make life a little more complicated [1,2].

Health status can affect one’s life routine and quality of life (QOL), especially when the health problem is chronic, and the treatment consumes time, as seen in patients with hemodialysis (HD) and peritoneal dialysis (PD) [2]. Many efforts have been made to improve the survival and QOL of patients with CKD through health education and promotion of awareness about the disease, rehabilitation programs for ESRD patients to assist them in leading a productive life, the use of erythropoietin-stimulating agents to correct anemia and improve QOL, proper management of comorbidities like cardiovascular diseases, developments of HD technology, the use of nocturnal and home HD, improved solute clearance, social and psychological support, reduced medication requirements, and proper management of malnutrition. These efforts would provide life-sustaining dialysis therapy and result in patients returning to full, active, and productive lives, including a return to employment [1,2].

PD is usually done at home, using either continuous cyclic PD with exchanges occurring at night for eight hours using a PD machine or continuous ambulatory PD with three to four exchanges a day. HD is usually done in an HD unit or in a hospital three times a week, with each session lasting four hours. Previous publications that compared both modalities showed that patients on HD or PD treatment were found to experience QOL deficits compared to general people. Even among these treatment modalities, the QOL varies from country to country. It is often dictated by the healthcare facilities provided by the country itself and the ease of accessing the variant medical services [3].

Several studies have reported the advantages of PD in some domains of QOL [4-10], the advantages of HD [11,12], and no differences between the two dialysis modalities [1,13]. In a Taiwanese study, the authors stated that PD patients tended to have a better QOL in terms of mobility and physiological conditions than HD patients [9]. Griffin et al. compared the QOL and the severity of illness between groups and found that HD patients appeared to suffer from physical symptoms to a greater degree than PD patients [10,14]. Meanwhile, in one study from Saudi Arabia, HD patients showed better psychological adjustment along several dimensions when compared to PD patients [13].

Our study aimed to compare the QOL between HD and PD patients in Bahrain. Given what is known about our population reports' culture, religion, and other social characteristics, we will compare the findings to previous national and international reports.

## Materials and methods

This cross-sectional study was conducted between the period from May to July 2023, targeting patients on HD or PD at Salmaniya Medical Complex (SMC), Bahrain. We recruited 90 HD patients and 40 PD patients; however, only 76 and 38 of them have been analyzed, respectively. Our inclusion criteria involved adults over the age of 18 who have gone through dialysis for at least three months, have not changed their treatment modality in the past three months, and do not have severe comorbidities, such as multi-organ failure, malignancies, stroke, and chronic liver disease or cognitive dysfunction. Initially, ethical approval was obtained from the Ethical Committee of the Institutional Review Board at Arabian Gulf University (E32-PI-2-23: 32451) and SMC (E32-PI-3-23: 17070323). All participants have been informed of the confidentiality of this study and that participation is voluntary. A verbal and written consent was obtained from all participants before obtaining any information, such as their demographic data, marital status, education level, occupation, duration on dialysis, cause of ESRD, social and exercise habits and traveling, any associated comorbidities, and any past hospital admissions and their causes. We used a structured questionnaire for collecting data and the Quality of Life Inventory (QOLI®) questionnaire for scoring. For every patient, we advised them to answer the questionnaire by themselves; however, for less educated patients, the investigators assisted in reading the questionnaire and filling out their answers.

QOLI description

QOLI is a multidimensional, validated questionnaire designed for patients on dialysis. It was developed by Ferrans and Powers [15] to measure both the importance and satisfaction of various aspects of life. Through the use of important ratings, we were able to assess the satisfaction responses in which scores reflect the respondents’ satisfaction with each aspect of life. Depending on the importance of every item, we gave a different score, in which essential items had a more significant impact on the scores and vice versa for less critical items. Through the following domains, i.e., health and functioning, psychological/spiritual, social and economic, and family, the overall QOL scores were calculated. The QOLI scores ranged from 0 to 30, with 30 being the highest score; however, for an easier and more accurate comparison of the two tools, the score has been transformed into a 0-100 score, with 100 being the highest score.

Statistical analysis

We used an Excel spreadsheet for the data entry and verification, which we exported into the IBM SPSS Statistics for Windows, version 28 (released 2021; IBM Corp., Armonk, New York, United States). We presented categorical variables as frequency and percentages and quantitative variables as mean ± standard deviation (SD). We used regression analysis to assess the linear relationship between sociodemographic (gender, age, marital status, level of education, and working status) and ESRD-related variables (dialysis duration and comorbidity type). A one-way analysis of variance method and Student’s t-test were used to compare the differences in QOL scores. We used Pearson's correlation coefficient to assess the interdomain correlation and the correlation between various demographic variables and domain scores. Statistical differences were considered significant if the p-value <0.05. 

## Results

A total of 114 respondents were included in this study. Out of 114 patients included in our survey, 76 patients were on HD and 38 patients were on PD (Table 1).

**Table 1:** Sociodemographic characteristics in hemodialysis (N = 76) and peritoneal dialysis (N = 38) groups Data represented as N and %. Differences with p < 0.05 were considered statistically significant. ESRD: end-stage renal disease

Characteristics		Hemodialysis (N = 76)	Peritoneal dialysis (N = 38)	Total (N = 114)	P-value
		N (%)	N (%)	N (%)	
Gender	Male	41(53.9)	21(55.3)	62 (54.4)	0.527
	Female	35 (46.1)	17 (44.7)	52 (45.6)	
Age groups	≤50 years old	16 (22.9)	10 (26.3)	26 (24.1)	0.714
	>50 years old	54 (77.1)	28 (73.7)	82 (75.9)	
Marital status	Single	11 (14.5)	4 (10.6)	15 (13.2)	0.599
	Married	55 (72.4)	30 (78.9)	85 (74.6)	
	Divorced	6 (7.9)	1 (2.6)	7(6.1)	
	Widow/Widower	4 (5.2)	3 (7.9)	7 (6.1)	
Education level	Illiterate	6 (7.9)	2 (5.3)	8 (7.0)	0.119
	High school or less	57 (75.0)	23 (60.5)	80 (70.2)	
	University education	13 (17.1)	13 (34.2)	26 (22.8)	
Causes of ESRD	Diabetes mellitus	33 (43.4)	6 (15.8)	39 (34.3)	0.758
	Hypertension	24 (31.6)	3 (7.9)	27 (23.7)	
	Glomerulonephritis	2 (2.6)	1 (2.6)	3 (2.6)	
	Polycystic kidney disease	4 (5.3)	1 (2.6)	5 (4.5)	
	Unknown	13 (17.1)	27 (71.1)	41 (35.9)	

More than 54% (n = 62) were males, with no statistical difference between the groups (p = 0.527). The mean age was 57.8 ± 11.3 years (58.7 ± 11.2 and 55.9 ± 12.1 years in the HD and PD groups, respectively), and 75.9% (n = 87) were ≥50 years in the two groups with no statistical difference (p = 0.327). The median duration of the dialysis was 2.5 years, and the mean duration of dialysis was 3.9 ± 2.9 years. Among the studied patients, 70.2% (n = 80) had a high school diploma or less, and 74.6 (n = 85) were married, with no statistical difference between groups. Diabetic mellitus and hypertension were the common causes of ESRD (n = 86, 58%). Table 1 shows the demographic characteristics of the study groups.

The number of hospital admissions was statistically significantly higher in the HD group (p = 0.007); however, there was no significant difference in the causes of admissions (p = 0.131). There was no significant difference in comorbidities during dialysis between the groups (p = 0.797), with diabetes mellitus and hypertension being the most associated comorbidities. PD patients could tolerate vigorous exercises compared to HD patients (p = 0.001). The clinical data of the study groups are shown in Table 2.

**Table 2:** Clinical data in the hemodialysis and peritoneal groups Data represented as N and %. Differences with p < 0.05 were considered statistically significant and p < 0.001 were highly significant.

Characteristics		Hemodialysis (N = 76)	Peritoneal dialysis (N = 38)	Total (N = 114)	P-value
		N (%)	N (%)	N (%)	
Comorbidities during dialysis	Hypertension	5 (6.6)	1 (2.6)	6 (5.3)	0.797
	Coronary artery disease/heart failure	2 (2.6)	0	2 (1.8)	
	Diabetes mellitus + hypertension	13 (17.1)	5 (13.2)	18 (15.8)	
	Diabetes mellitus	1 (1.3)	1 (2.6)	2 (1.8)	
	Others	2 (2.6)	0	2 (1.8)	
	None	53 (69.7)	31 (81.6)	84 (73.7)	
Exercise tolerance	Light	28 (36.8)	6 (15.8)	34 (29.8)	0.001
	Vigorous	1 (1.4)	3 (7.9)	4 (3.5)	
	N/A	47 (61.8)	29 (76.3)	76 (66.7)	
Admission to the hospital	0	36 (47.4)	25 (65.8)	61 (88.6)	0.007
	1-2	26 (34.2)	5 (13.1)	31 927.2)	
	3-4	7 (9.2)	2 (5.3)	9 (7.9)	
	More than 4	7 (9.2)	6 (15.8)	13 (11.3)	
Causes of admission	Infection/sepsis	3 (3.9)	3 (7.9)	6 (5.3)	0131
	Ketoacidosis	1 (1.3)	-	1 (0.9)	
	Access problem	5 (6.6)	-	5 (4.4)	
	Cardiac causes	4 (5.3)	1 (2.6)	5 (4.4)	
	Other	25 (32.9)	3 (7.9)	28 (24.7)	
	No.	38 (50.0)	31 (81.6)	69 (60.5)	

In this study, we noted that the family subscale has the highest QOL score (93.2 ± 9.2 and 98.6 ± 4.7), followed by the psychological/spiritual subscale (81.1 ± 16.7 and 97.6 ± 3.9) in the HD and PD groups, respectively. Still, it was statistically significantly higher in the PD group (p < 0.001) (Table 3). Most patients on PD had faith in God and were happy with their lives, while patients on HD had faith in God in their lives but were satisfied with their family and children's health. The mean scores in each category of the domains are shown in Table 3.

**Table 3:** Comparison of the mean scores of specific dimensions of QoL among the hemodialysis and peritoneal dialysis patients Data represented as mean ± SD. Differences with p < 0.05 were considered statistically significant and p < 0.001 were highly significant.

Dimensions	Hemodialysis	Peritoneal dialysis				
	Mean	SD	Mean	SD	P-Value	
Health and functioning	78.25	13.47	93.27	5.95	< 0.001	
Your health?	96.49	9.55	99.12	3.77	0.105	
Your health care?	95.11	10.53	98.25	6.47	0.096	
The amount of energy you have for everyday activities?	76.22	28.37	98.25	6.47	< 0.001	
Your ability to take care of yourself without help?	78.22	27.40	99.10	3.82	< 0.001	
The likelihood you will get a kidney transplant?	69.52	37.16	75.00	31.18	0.436	
The changes you have had to make in your life because of kidney failure (such as diet and need for dialysis)?	81.78	24.07	90.54	14.98	0.045	
The amount of control you have over your life?	84.67	17.28	94.44	14.36	0.004	
Your family’s happiness?	98.65	4.58	100.00	0.00	0.076	
The emotional support you get from people other than your family?	73.90	28.72	91.67	16.33	0.001	
Your ability to take care of family responsibilities?	74.32	30.62	98.20	5.25	< 0.001	
How useful you are to others?	77.56	27.05	96.40	10.49	< 0.001	
How well you can take care of your financial needs?	86.94	16.38	95.05	14.63	0.012	
The things you do for fun?	71.33	32.60	87.84	19.50	0.005	
Social and Economic	73.12	15.47	84.11	14.24	< 0.001	
Your spouse, lover, or partner?	78.36	32.12	94.62	16.88	0.010	
The emotional support you get from your family?	87.56	18.80	98.65	6.06	0.001	
The amount of worries in your life?	86.89	20.00	97.37	7.28	0.002	
Your neighborhood?	68.86	30.35	85.14	21.80	0.004	
Your home, apartment, or place where you live?	95.11	12.49	99.12	3.77	0.056	
Your job (if employed)?	55.56	40.43	72.22	41.67	0.320	
Not having a job (if unemployed, retired, or disabled)?	45.24	34.98	44.44	28.64	0.937	
Your education?	46.32	30.86	48.00	32.39	0.824	
Psychological/spiritual	81.08	16.65	97.56	3.85	< 0.001	
Your chances for a happy future?	77.70	25.17	99.52	2.82	< 0.001	
Your peace of mind?	86.26	20.34	100.00	0.00	< 0.001	
Your faith in God?	97.59	9.70	100.00	0.00	0.129	
Your achievement of personal goals?	62.72	33.65	93.81	15.17	< 0.001	
Your happiness in general?	86.40	19.57	99.56	2.70	< 0.001	
Your life in general?	85.75	18.80	100.00	0.00	< 0.001	
Your personal appearance?	62.06	34.92	89.04	19.48	< 0.001	
Yourself in general?	87.28	20.52	100.00	0.00	< 0.001	
Family	93.19	9.18	98.60	4.69	0.001	
Your chances of living as long as you would like?	76.10	27.40	92.59	13.48	0.001	
Your family’s health?	98.25	6.43	99.12	5.41	0.446	
Your children?	98.41	5.77	99.51	2.86	0.301	
Your sex life?	34.29	27.10	69.57	26.90	< 0.001	
Your friends?	81.56	25.93	85.96	21.76	0.343	
Quality of life	79.78	11.71	93.11	5.66	< 0.001	

Table 4 and Table 5 show details of Pearson’s correlations among various domains in the HD (Table 4) and PD (Table 5) groups. The Person's correlation coefficient indicates that the correlation coefficient is highly significant between the domain scores except between the family and psychological/spiritual domains (p-value = 0.101) in the HD group family and social and economic, psychological/spiritual domains (p = 0.052 and 0.089, respectively) in the PD group. When we analyzed the strength of association among various domains, we showed a moderate interdomain correlation between the family domain and total QOL score, health and functioning, and psychological/spiritual domains (Pearson’s r > 0.3 and < 0.5) in the HD group and between the family domain and social and economic and psychological/spiritual domains in the PD group. The other associations were strong (Pearson’s r > 0.5).

**Table 4:** Pearson's correlation coefficient between the domain scores for the hemodialysis group Data represented as Pearson's correlation coefficient. Differences with p < 0.05 were considered statistically significant and p < 0.001 were highly significant.

		Quality of life	Health and functioning	Social and economic	Psychological/spiritual	Family
Quality of life	Pearson's correlation	1				
	P-value					
Health and functioning	Pearson's correlation	0.931	1			
	P-value	<0.001>				
Social and economic	Pearson's correlation	0.786	0.596	1		
	P-value	<0.001>	<0.001>			
Psychological/spiritual	Pearson's correlation	0.838	0.751	0.471	1	
	P-value	<0.001>	<0.001>	<0.001>		
Family	Pearson's correlation	0.477	0.32	0.531	0.19	1
	P-value	<0.001>	0.005	<0.001>	0.101	

**Table 5:** Pearson's correlation coefficient between the domain scores for the peritoneal dialysis group Data represented as Pearson's correlation coefficient. Differences with p < 0.05 were considered statistically significant and p < 0.001 were highly significant.

		Quality of life	Health and functioning	Social and economic	Psychological/spiritual	Family
Quality of life	Pearson's correlation	1				
	P-value					
Health and functioning	Pearson's correlation	0.844	1			
	P-value	<0.001>				
Social and economic	Pearson Correlation	0.860	0.517	1		
	P-Value	<0.001>	<0.001>			
Psychological/spiritual	Pearson's correlation	0.613	0.39	0.475	1	
	P-value	<0.001>	0.015	0.003		
Family	Pearson's correlation	0.619	0.596	0.317	0.28	1
	P-value	<0.001>	<0.001>	0.052	0.089	

Table 6 shows the QOL score and domains in relation to age between the two studied groups. The results show that age did not affect the high QOL scores in the PD group compared to the HD group in all domains (p < 0.001). However, in Table 7, which shows the QOL in relation to the duration of dialysis between the two groups, the scores were not statistically significantly higher (except in the social and economic domain) in the PD group compared to the HD group in patients on regular dialysis <one year, while in patients on regular dialysis >one year, the scores were statistically significantly higher in the PD group.

**Table 6:** Comparison between dialysis duration groups for both groups Data represented as mean ± SD. Differences with p < 0.05 were considered statistically significant and p < 0.001 were highly significant.

	One year					More than one year				
	Hemodialysis		Peritoneal dialysis		P-value	Hemodialysis		Peritoneal dialysis		P-value
	Mean	SD	Mean	SD		Mean	SD	Mean	SD	
Quality of life	80.68	11.76	90.56	1.2	0.421	79.65	11.70	93.83	10.28	0.005
Health and functioning	78.78	14.20	92.31	2.1	0.362	78.13	13.18	92.58	10.40	0.004
Social and economic	75.31	14.53	70.00	1.3	0.724	72.56	16.26	92.50	16.98	0.001
Psychological/spiritual	82.22	15.94	95.24	1.1	0.433	81.27	17.21	96.94	4.70	0.01
Family	92.79	8.69	100.00	1.0	0.426	93.36	9.65	95.71	8.76	0.265

**Table 7:** Comparison between younger and older patients in both groups Data represented as mean ± SD. Differences with p < 0.05 were considered statistically significant and p < 0.001 were highly significant.

	Younger group					Older group				
	Hemodialysis		Peritoneal dialysis		P-value	Hemodialysis		Peritoneal dialysis		P-value
	Mean	SD	Mean	SD		Mean	SD	Mean	SD	
Quality of life	83.26	11.93	96.96	3.86	0.002	79.00	11.49	91.73	5.61	<0.001
Health and functioning	84.12	12.16	98.09	2.32	0.002	76.62	13.38	91.55	5.92	<0.001
Social and economic	76.20	15.93	92.38	11.54	0.006	72.60	15.66	81.16	14.11	0.015
Psychological/spiritual	84.28	19.11	97.86	4.55	0.038	80.76	16.04	97.45	3.66	<0.001
Family	92.50	7.37	99.67	1.05	0.006	93.38	9.85	98.21	5.40	0.018

In Table 8, the regression analysis shows that QOL is inversely related to gender, age, and comorbidities during dialysis and positively related to marital status and educational level. All demographic variables were not statistically significant (p-value > 0.05).

**Table 8:** Regression analysis between the quality of life and the demographic factors for the HD and PD groups Data represented as factor, coefficient, and corresponding p-values.

	Coefficient	Std. error	Beta	T	p-value
(Constant)	105.25	13.18		7.98	0.00
Gender	-4.65	4.55	-0.23	-1.02	0.32
Marital status	1.05	3.42	0.07	0.31	0.76
Education level	4.65	4.41	0.25	1.05	0.30
Age	-0.25	0.17	-0.30	-1.45	0.16
Comorbidities during dialysis	-0.43	0.38	-0.25	-1.12	0.28

## Discussion

Renal replacement therapy for patients with ESRD, such as PD or HD, can improve QOL. QOL is a significant predictor of patient outcome and survival after starting HD or PD. Thus, it is essential to assess the QOL of ESRD patients and take the necessary steps to improve their living conditions. The results of our study show that patients on PD had better QOL than patients on HD, which is consistent with most of the published studies [4-10]. A study from Saudi Arabia found that the mean scores of QOL were significantly higher among PD in all domains except for the physical QOL score, which was non-statically significantly higher in HD patients than PD patients [8]. Our results show that patients with PD, compared to HD patients, reported a better QOL in all domains except social and economic relationships, which may indicate difficulties in employment in both groups. Accordingly, both groups experienced less support from their community and social relationships. 

In our study, we observed that the highest score (98.6 ± 4.7 and 93.2 ± 9.2) was seen in the family domain, followed by the psychological/spiritual domain (97.6 ± 3.9 and 81.2 ± 16.7) in PD and HD, respectively. High scores (>90) were observed only in the "faith in God” subcategory of the psychological/spiritual subscale, which may be related to religious beliefs among Arabic Muslim people. This was supported by the argument that Arab and Muslim people receive considerable support from their families when they become ill [7,16]. Low scores (<60) were seen in the "work status" and "sexual life" subcategories in both groups. We could explain this to the fact that most of the studied patients had chronic comorbidities, and most of them were currently not working or had difficulties in their jobs. Our study also shows that most of the patients in the PD group are satisfied with their social life (score > 90) and their relationship with families, which indicates strong ties in the families and communities in Arabic countries, and this is shown in many other studies [7,16-18]. HD patients have lower scores and this may be related to the restrictions imposed on their lives and the dependence on the dialysis procedure, which may cause social isolation and problems with transportation to their unit and waiting time before and after dialysis sessions [18,19].

Our results show that patients with PD, whether older or younger, have better QOL in all domains compared to HD patients. Being older explains why HD patients score less on physical activity-related items. However, this could not explain the better QOL in PD patients in all other domains. The age gap between HD and PD patients has been observed in previous studies [18,19]. These differences, collectively, can be explained by the possibility that PD programs generally select younger, more educated, healthier patients with more strong relationships with friends and families, which may contribute to the lower response rate observed among HD patients [18,20].

Patients on dialysis for less than one year show a non-significant difference in QOL scores between both groups, although it is higher in PD patients. At the end of one year, patients with PD are still feeling less burdened by the disease itself. Higher satisfaction with their social life and less burden of the dialysis procedure was sustained in patients on PD for a longer time because they are freer and able to enjoy more social life compared to HD patients since patients on HD need longer hours of hospital stay for dialysis, which can harm their personal and professional lives. Similar results with respect to the advantage of PD regarding dialysis duration and treatment satisfaction were shown in different studies [7,21,22].

The regression analysis indicates that the QOL is inversely related to gender, age, and comorbidities during dialysis and positively related to marital status and educational level, although they were not statistically significant. These results agree with another study from Saudi Arabia [7]. We could explain that age, male gender, and comorbidities on dialysis are negative predictors of QOL scores by the higher percentage of male patients in both groups and female patients are not self-dependent.

The limitations of the study may include the following: (1) The sample size is relatively small. (2) There was no information on measured pre-dialysis QOL, which might have yielded more useful results. (3) Considering the international variation in QOL, caution should be taken when applying our findings to other ethnicities because we included only Arab patients. (4) We had no information about the primary reason for initiating the dialysis modality of choice, which can be affected by social and medical contraindications except for patients' own modality choice.

## Conclusions

Our findings show that patients with PD have better QOL scores in all domains than patients with HD. PD patients were burdened less by ESRD symptoms and were able to continue their jobs and social life. Unlike patients with PD who perform dialysis at home, patients with HD have to go to dialysis units three times a week for four hours per session, and this negatively affects both their social lives and occupational attainment. Moreover, patients receiving PD maintained social support and ultimately felt better general physical health and emotional well-being. In this study, after one year, patients on PD are still feeling less affected by the disease burden. Our results may represent essential information for patients who are reluctant to initiate PD treatment modality.
